# Species richness and macronutrient content of *wawo* worms (Polychaeta, Annelida) from Ambonese waters, Maluku, Indonesia

**DOI:** 10.3897/BDJ.3.e4251

**Published:** 2015-02-27

**Authors:** Joko Pamungkas

**Affiliations:** ‡ Research Center for Deep Sea, Indonesian Institute of Sciences, Ambon, Indonesia

**Keywords:** *Wawo* worms, species richness, macronutrient content, Polychaeta, Ambonese waters

## Abstract

The aims of this research were to: (1) investigate the species richness of *wawo* worms, and to (2) analyze macronutrient content of the worms. *Wawo* worms were sampled using a fishing net on March 18^th^-19^th^, 2014, from Ambonese waters, Maluku. As many as 26 *wawo* species belonging to 5 families were identified. *Palola* sp. was identified as the most abundant species of *wawo*, followed by *Lysidice oele*, Horst 1905, *Eunice* spp. and nereidids. Results of the proximate analysis reveal that female epitokes of *Palola* sp. contain 10.78 % ash, 10.71 % moisture, 11.67 % crude fat, 54.72 % crude protein and 12.12 % carbohydrate.

## Introduction

Similar to Pacific *palolo*, *wawo* or *laor* are edible marine worms (Polychaeta, Annelida) that are consumed by natives of Ambon, Maluku. These animals swarm twice a year to reproduce, i.e. either in February and March (Rumphius 1705), or in March and April (Radjawane 1982). The worms swarm exclusively in Ambonese coastal waters with reefs, either in the form of epigamous epitokes (i.e. sexually mature worms with heads; adapted to swim) or schizogamous epitokes (also called 'stolon', i.e. headless sexually mature worms; adapted to swim). The swarming phenomenon, the tradition of catching *wawo* (called ‘*timba laor*’ among the locals) and the recipe of the traditional dish, are reported in detail by Pamungkas 2011 (Fig. 1).

To date, the species richness of *wawo* remains uncertain. This is because different scientists studied in different areas. For instance, Horst 1904, Horst 1905 obtained *Lysidice oele* (family Eunicidae) from Banda Islands whereas Martens et al. 1995 identified a mix of 13 different *wawo* species (5 families) from Ambonese waters (Airlouw Village). This, consequently, makes the study incomparable. Among locals, *wawo* worms are also considered a nutritious dish due to their high protein content, but no scientific publication has confirmed this assumption yet.

The aims of this study were to: (1) identify the species richness of *wawo* worms, and to (2) analyze macronutrient contents of the worms.

## Material and methods

**Figure 2.**
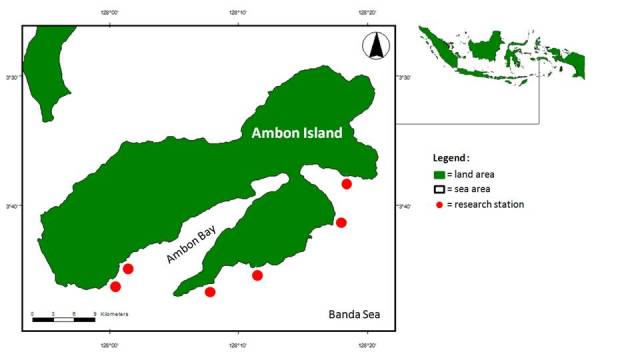
Map of the research stations. Clockwise (starting from the lower left): station 1, 2, 3, 4, 5 and 6.

During the swarming time of *wawo* on March 18^th^-19^th^, 2014, *wawo* were sampled (Fig. 2). In this study, the author hypothesizes that more stations will yield more *wawo* species. The animals were caught in a fishing net (Ambonese: *siru-siru*) and were immediately fixed with 10% formaldehyde solution for about 24 hours. They were then rinsed with tap water and were further preserved in 70% ethanol. The specimens were identified under stereo and compound microscopes. Photomicrographs were taken using a DSLR camera attached to the stereo microscope. Due to a lack of taxonomic information on Indonesian polychaetes, some of *wawo* species can not be identified to species level. *Wawo* specimens obtained in this study are deposited at the *Museum Zoologicum Bogoriense* (MZB), Bogor, Indonesia, and at the Reference Collection LIPI Ambon (RCLA) which belong to the Research Center for Deep Sea, Indonesian Institute of Sciences (LIPI), Indonesia.

The proximate analysis, namely a quantitative analysis of a compound to determine the percentage of its constituents (i.e. ash, moisture, crude fat, crude protein and carbohydrate) was also applied to obtain information on *wawo*’s macronutrient. Female epitokes of *Palola* sp. were selected as samples for the analysis, and were collected from Alang waters on March 14^th^, 2009. The analysis was done at the Faculty of Fisheries and Marine Sciences, University of Pattimura, Ambon, with methods referring to Horwitz 1980.

Ash was measured with the following procedure. An empty porcelain cup was first heated in a furnace at a temperature of 600˚C, cooled in a desiccator until room temperature is reached, and weighed (W1). The *wawo* sample (2 grams; wet weight; W2) was then placed on the cup. The cup with the sample was further heated to 600˚C, cooled in a desiccator until room temperature is reached, and weighed (W3). The heating process was repeated for half an hour until constant weight is reached. The following equation is used to calculate ash: (W3-W1)/ W2 x 100%.

Moisture was measured with the following procedure. An empty petri dish was first heated in an oven at a temperature of 105˚C for 3 hours, cooled in a desiccator until the room temperature is reached, and weighed (W1). The *wawo* sample (2 grams; wet weight; W2) was then placed on the dish. The dish with the sample was further heated in an oven at a temperature of 105˚C for 3 hours, cooled in a desiccator until the room temperature is reached, and weighed (W3). The heating process was repeated for several times until constant weight of sample is reached. The following equation is used to calculate moisture: (W3-W1)/ W2 x 100%.

Crude fat and protein were measured using Soxhlet and Kjeldahl method, respectively (Horwitz 1980), with 2 grams of dry sample were used for each analysis. In this study, carbohydrate was not directly measured, but was estimated by calculating the so-called 'nitrogen-free extract' (NFE) using the following equation: NFE = 100% - (Ash + Moisture + Fat + Protein).

## Results and Discussion

As many as 25 different species of *wawo* were discovered, including 3 species in the form of schizogamous epitokes and 22 species in the form of epigamous epitokes. The epigamous epitokes consist of 5 families, i.e. Eunicidae (7 species), Euphrosinidae (1 species), Lumbrineridae (3 species), Nereididae (9 species) and Scalibregmatidae (2 species) (Table 1). The presence of *Euphrosine* sp. (Euphrosinidae) among swarming *wawo* is reported for the first time in this study. Two *wawo* species have been identified as potential new species, i.e. Neanthes cf. gisserana and *Nereis* sp. (Glasby, pers. comm.).

The most abundant *wawo* species at most stations is *Palola* sp. These animals are headless, relatively thin and have either pale yellow or green colors for male and female animals, respectively (Fig. 3b). Despite the absence of heads, the animals possess the same characteristics of both chaetae and parapodia of *Palola viridis* Gray, 1847, as described by Martens et al. 1995 in their publication. Also, the female ones agree well the description of Stair 1847: “Green, with a row of round black spots down the middle of the dorsal ? surface; one spot on the middle of each joint”. Nevertheless, the scientific name *Palola* sp. is preferably used here (than *P. viridis*) as the head of the worms as the primary character to define species is completely absent.

*Palola* sp. is also known as the islanders’ favourite as they smelt easily when sauted on a wok and thereby no longer look like worms. By contrast, bigger *wawo* that annually swarm in April are caught by only a few of locals due to their more unappetizing appearance. The later group of *wawo*, interestingly, has not been studied yet by scientists.

**Figure 4.**
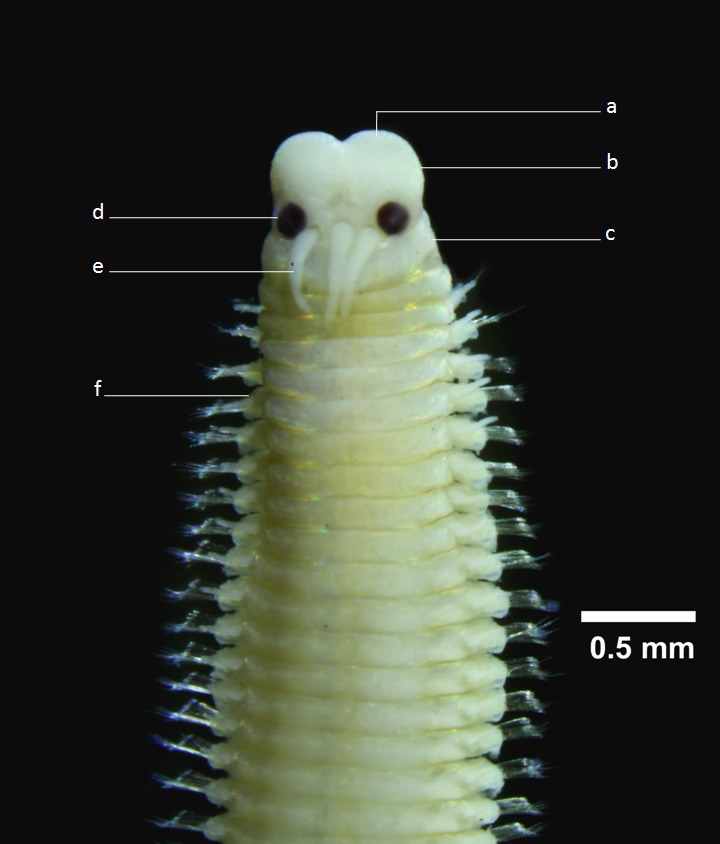
*Lysidice oele* Horst, 1905. a=palp; b=prostomium; c=peristomium; d=eye; e=occipital antenna; f=parapodium with chaetae.

In this study, *Lysidice oele* Horst, 1905 (Fig. 4) is the second common species of *wawo* and was present at all stations, followed by *Eunice* spp. (both of them belong to the family Eunicidae – Table 1). Along with members of the family Nereididae, they are well-known for their reproduction strategy called epitoky, i.e. a phenomenon wherein mature adults modify their bodies into swimming forms (epitokes) and swarm in the water column to spawn (e.g. see Baoling et al. 1985; Caspers 1984; Chatelain et al. 2007; Woodworth 1907).

The study also indicates that different stations generated different *wawo* species. This is most likely due to variations in habitat characteristics like differences in types, distribution and healthiness of coral reefs among stations. Nevertheless, this requires further investigations to prove. Differences in species richness among stations might also be due to variations in sampling effort.

Species number of *wawo* found in the present study (25 species) is higher than that of Martens and Horst’s studies (13 and 1 species, respectively). This supports the author's hypothesis that more study sites will yield more species of *wawo* due to differences in habitat characteristics. This also shows how diverse *wawo* are. In fact, *wawo* are also present in several different sites in Maluku waters such as Banda, Haruku, Nusalaut, Pombo, Saparua and Tual waters, but most of them are poorly or even unstudied (pers.obs.). This means that if we sample the animals in those unstudied sites, we are likely to discover both new species and new record species of *wawo*.

Besides high in species richness, *wawo* are also nutritious with 54.72% of their body is protein (Table 2). The crude protein (54.72%), the carbohydrate (12.12%) and the crude fat (11.67%) of *wawo* in this study are higher than those of Radjawane’s findings (i.e. 31.15%, 0.41% and 7.76%, respectively). The results difference between present and past study is due to, first, different method of analysis, and, second, the fact that Radjawane analyzed a mix of several different species of *wawo* (including male and female epitokes), whereas in this study only female epitokes of *Palola* sp. were analyzed. High percentage of *wawo* protein is due to the presence of gamets (either sperm or ovum) filling their bodies when the worms swarm.

### Conclusion

It is obvious from the study that the species richness of Ambonese *wawo* was considerably higher than that of previous studies as more research stations were included in the present study. This indicates how diverse *wawo* are, more than what has been known for decades. The study also confirms the locals' assumption that *wawo* are high in protein.

## Figures and Tables

**Figure 1.** *Timba laor* (video). The video shows how natives of Ambon catch and cook the worms.

**Figure 3a.**
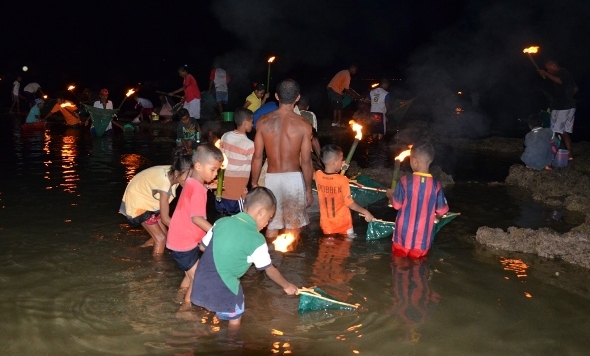
Natives of Ambon were catching *wawo.*

**Figure 3b.**
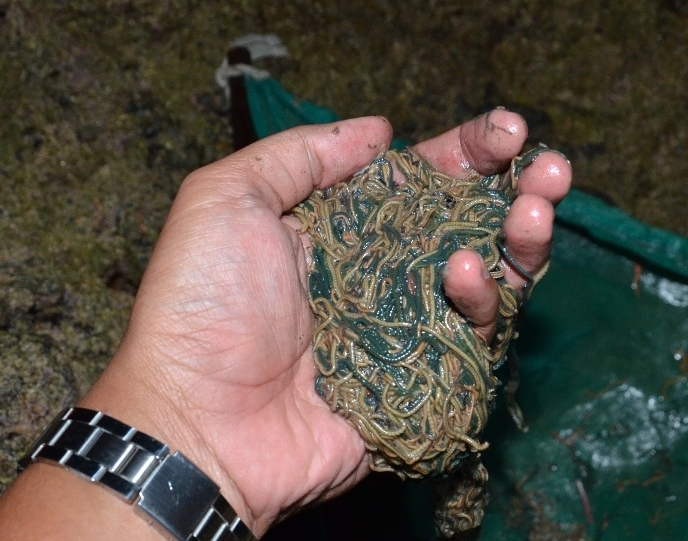
*Palola* sp. - the common species of *wawo*.

**Table 1.** Species list of *wawo*.

**Types of Epitokes**	**Species**	Stations*					
		**1**	**2**	**3**	**4**	**5**	**6**
**Epigamous**							
	**Family Eunicidae**						
	*Eunice* sp. 1	+	+	+	-	+	-
	*Eunice* sp. 2	+	+	+	-	+	-
	*Eunice* sp. 3	+	+	-	-	-	-
	*Eunice* sp. 4	-	+	+	-	+	-
	*Eunice* sp. 5	-	+	-	-	-	-
	*Eunice* sp. 6	-	+	-	-	+	-
	*Lysidice oele* Horst, 1905	+	+	+	+	+	+
	**Family Euphrosinidae**						
	*Euphrosine* sp.	-	+	+	+	+	+
	**Family Lumbrineridae**						
	*Lumbrineris* sp. 1	+	+	+	-	+	+
	*Lumbrineris* sp. 2	-	+	+	-	+	-
	*Lumbrineris* sp. 3	-	+	-	-	-	-
	**Family Nereididae**						
	*Ceratonereis singularis australis* Hartmann-Schröder, 1985	-	-	+	-	-	-
	*Composetia marmorata* (Horst, 1924)	-	-	+	-	+	-
	Neanthes cf. gisserana (Horst, 1924)	-	-	+	-	+	-
	*Neanthes masalacensis* (Grube, 1878)	-	-	+	-	-	-
	*Neanthes unifasciata* (Willey, 1905)	+	+	+	-	+	-
	*Nereis* sp.	-	-	+	-	+	-
	*Pereinereis helleri* (Grube, 1878)	+	-	-	+	+	+
	*Perinereis nigropunctata* (Horst, 1889)	+	-	+	+	+	-
	*Solomononereis marauensis* Gibbs, 1971	-	-	+	-	-	-
	**Family Scalibregmatidae**						
	*Hyboscolex verrucosa* Hartmann-Schröder, 1979	-	-	-	-	-	+
	Scalibregmatidae sp.	-	-	-	-	-	+
**Schizogamous/ Stolon**							
	**Family Eunicidae**						
	*Palola* sp.	+	-	+	+	+	+
	Eunicidae sp. 1	-	+	-	-	-	-
	Eunicidae sp. 2	+	-	+	+	-	+
	**Total Number of Species**	10	13	17	6	15	8

**Table 2.** Proximate analysis results (in % of weight)

**Ash**	**Moisture**	**Crude Fat**	**Crude Protein**	**Carbohydrate**
10.78	10.71	11.67	54.72	12.12
